# Stakeholder perspectives on digital wellbeing in Saudi Arabia: a cross-sectional survey

**DOI:** 10.1186/s12889-025-23674-4

**Published:** 2025-07-14

**Authors:** Saud Abdulaziz Alomairah, Michelle Colder Carras, Dahlia Aljuboori, Juliann Saquib, Nazmus Saquib, Mayank Date, Kyeongwon Kim, Faisal Aldayel, Michelle R. Kaufman, Laura K. Clary, Vincent G. van der Rijst, Anouk Tuijnman, Antonius J. van Rooij, Nahlah Alsogaih, Faisal Z. Al-Zabidi, Fahad S. Albeyahi, Johannes Thrul

**Affiliations:** 1 Department of Mental Health, Johns Hopkins Bloomberg School of Public Health, 615 N. Wolfe Street, Baltimore, 21205 USA; 2 Department of International Health, Johns Hopkins Bloomberg School of Public Health, Baltimore, MD USA; 3 Sulaiman Alrajhi University, Qassim, Saudi Arabia; 4 Department of Health, Behavior and Society, Johns Hopkins Bloomberg School of Public Health, Baltimore, MD USA; 5 Ministry of Health, Riyadh, Saudi Arabia; 6 Trimbos Institute, Utrecht, The Netherlands; 7 King Abdulaziz Center for World Culture (Ithra), Dhahran, Saudi Arabia; 8 Sidney Kimmel Comprehensive Cancer Center at Johns Hopkins, Baltimore, MD USA; 9 Centre for Alcohol Policy Research, La Trobe University, Melbourne, Australia

**Keywords:** Digital wellbeing, Internet, Stakeholder engagement, Health promotion, Survey, Digital balance

## Abstract

**Background:**

In Saudi Arabia, the rapid growth of digital media and smartphone use has raised concerns about problematic usage and its impacts on well-being, especially among young people. Research on stakeholder perspectives regarding intervention strategies remains limited.

**Objective:**

This study aimed to gather insights from societal stakeholders, including youth, parents, policymakers, industry leaders, clinicians, educators, and digital media users, to inform culturally tailored interventions for digital well-being in Saudi Arabia.

**Methods:**

A purposeful non-random sample of 92 participants representing different stakeholder groups was recruited to complete an online survey, answering questions about their experiences and perspectives on digital media use. Primary stakeholder group was assigned based on participant self-selection. We analyzed distributions of categorical variables related to media use time, reasons for use, impacts, self-regulation strategies, and perceived effectiveness of interventions (e.g., education programs, media campaigns, Internet use restrictions).

**Results:**

Of the participants, 63.0% were male, and 46.7% were under 25 years old. Regular digital media users, individuals with problematic Internet use, and clinicians/health professionals comprised 26.1%, 18.5%, and 18.5% of respondents, respectively. Extensive screen time was common, with 47.8% reporting four or more hours of recreational digital use on weekdays and 56.6% on weekends. Participants reported both positive impacts (e.g., social connections, school/work performance) and negative impacts (e.g., sleep disruption, reduced physical activity) of digital media use. Efforts to regulate media use were reported by 72.8%, with strategies like deleting apps or digital detoxes. At least 50.0% of participants endorsed all proposed intervention approaches as likely effective for improving digital well-being, with educational programs for parents, school programs, and regulatory apps receiving over 75.0% support. Children and adolescents were seen as key target groups for these interventions.

**Conclusions:**

Findings from this diverse stakeholder sample suggest that digital well-being interventions in Saudi Arabia should prioritize youth, focusing on education-based approaches and apps for media regulation. Incorporating these perspectives can lead to culturally relevant interventions addressing the unique challenges of digital media use in Saudi Arabia. The generalizability of the findings may be limited due to sample size and potential overrepresentation of certain stakeholder groups.

**Supplementary Information:**

The online version contains supplementary material available at 10.1186/s12889-025-23674-4.

## Background

The growing use of and reliance on technology in Saudi Arabia [1] has led to concerns about problematic internet use, internet addiction, and negative impacts on mental health and wellbeing, especially among young people [2]. At the same time, however, there is recognition of the ubiquity of internet access, which is now part of everyday life for young people [3, 4]. Given that daily internet use is now the norm for many Saudi Arabians and problematic use is high [5, 6], addressing these concerns and developing interventions to promote digital wellbeing have emerged as critical priorities.

Digital wellbeing can be defined as the subjective individual experience of achieving a healthy balance in using digital media and technology while avoiding problems arising from excessive usage [7]. A 2018 systematic review of prevention approaches for digital wellbeing interventions summarized studies from 7 countries (not including Saudi Arabia) [8] and showed that these approaches vary depending on the country and region. These range from the treatment of addiction as a public health threat with a coordinated government response in East Asian countries to programs led mainly by nonprofit organizations or private entities in Western countries.

Internet access in Saudi Arabia is high [9], yet wide accessibility is more recent than in other countries, including neighboring countries [10]. Therefore, younger generations experience being constantly connected to the internet, in contrast with older generations [10]. In addition, the Saudi government’s *Saudi Vision 2030* [11] aims to diversify the economy and promote social and cultural reforms, emphasizing digital transformation and the digital economy. Notably, recent investments in the video game industry [12] and the launch of the Esports World Cup [13] mark this as a turning point in Saudi culture regarding technology use.

Recent studies on digital media use in Saudi Arabia have primarily focused on the prevalence and risk factors of problematic internet use/internet addiction [5, 14–16]. For example, in a nationally representative sample of Saudi adolescents aged 13 to 18 years, the prevalence of problematic internet use was 41.8%, with internet addiction rate of 3.7% [16]; with sex, immigration background, history of use, and family-related variables found to be potential risk factors. Addiction to smartphones is also a growing concern in Saudi Arabia. A study of sixth-year medical students in Jeddah reported a prevalence of smartphone addiction, defined as excessive use that disrupts daily life, of 36.5% [14], and a second study of university students in Riyadh found a prevalence of 48.0% [15]. Yet, few studies explore the cultural attitudes and societal factors contributing to digital well-being concerns [2, 16].

Knowing the influence of predisposing, reinforcing, and enabling factors such as cultural attitudes and beliefs is vital for understanding their influence on health behaviors and setting goals for population health interventions [17]. Considering various stakeholder perspectives is relevant for the development of successful digital wellbeing interventions [18, 19]. Stakeholder engagement allows researchers to understand the changing nature of technology as well as the cultural and societal contexts and can help ensure that interventions meet the needs of target populations [17, 20]. Despite the increasing acknowledgment of the need for culturally tailored interventions to promote balanced digital media use, notable gaps persist in the literature concerning this priority in Saudi Arabia [16].

To address these gaps, the current study sought to collect the opinions of various societal stakeholders (e.g., young people, parents, policy makers, industry leaders, clinicians, and educators) to inform the development of culturally tailored and grounded interventions to prevent problematic digital media use and promote digital wellbeing in Saudi Arabia. We conducted a survey to answer the following research questions:

- What are stakeholders’ own experiences with digital technology use and which strategies do they use to optimize their digital wellbeing?
- What types of interventions to improve digital wellbeing do stakeholders think would work in Saudi society and which population do they think would benefit most from intervention?

## Methods

### Procedure

Purposeful non-random sampling was used to select individuals in Saudi Arabia through known networks of Saudi investigators. We first identified potential stakeholder groups that could provide information relevant to developing digital wellbeing interventions, which was led by Saudi team members. After consultation with the broader research team, we developed a detailed list of criteria for participant inclusion, aiming to capture diverse perspectives within each identified group (Table 1).

**Table 1:** Characteristics of stakeholders (*n* = 92)

Stakeholder characteristic	Percent
**Sex**	
Male	63.0
Female	37.0
**Age Groups**	
18–24	46.7
25–34	26.1
35–44	9.8
45–54	12.0
55–64	5.4
**Highest Level of Education**	
Completed primary school (0–6 grade)	1.1
Completed intermediate school (7–9 grades)	10.0
Completed high school (10–12 grades)	39.1
Completed University/Technical Training	38.0
Post graduate degree	18.5
**Employment Status**	
Student	37.0
Employed (government, non-government, or self-employed)	42.4
Not employed	10.9
Homemaker	7.6
Other/retired	2.1
**Primary stakeholder group identity (Participants were asked to choose a single identity single answer, *n* = 92)**	
Not a regular user of digital media for entertainment	4.3
Regular user of digital media for entertainment	26.1
Other professional (*n* = 37)	
* Academic Researcher*	3.3
* Clinician/Healthcare professional*	18.5
* Educator*	6.5
* Policymaker*	5.4
* Religious leader, e.g., an imam*	2.2
* Judge or social worker in court*	1.1
* Member of the information and communication technology or game industry*	2.0
Gamer or professional gamer/social media influencer (*n* = 8)	
* Gamer*	7.6
* Pro gamer or social media influencer*	1.1
Person with experience of problematic internet use (*n* = 17)	
* Person with intensive and potentially problematic internet, social media, or game use*	18.5
* Family member/friend of a person with intensive and potentially problematic internet, social media, or game use*	2.2
I cannot answer	1.1
**Province**	
Qassim	45.7
Riyadh	25.0
Eastern province	9.8
Al-Medinah	4.3
Mecca	7.6
Other provinces	7.6
**Monthly Household Income (Saudi riyal)**	
Less than 7,000	29.3
7,000 to 20,000	21.7
More than 20,000	18.5
Other/don’t know	30.5
**Marital Status**	
Currently married	33.7
Never married	64.1
Other/I cannot answer	1.1
Divorced	1.1

Potential stakeholders were first provided with a link through WhatsApp to an online eligibility survey, which was used to categorize them by stakeholder type and secure contact information. The study team discussed selected members from each of the stakeholder groups and sent them the link to the full survey. The study team regularly reviewed the progress and composition of the sample who completed the survey and selected additional participants within each group to ensure comprehensive coverage across stakeholder groups. Once the target sample size had been reached, the recruitment was ended, and the survey link was closed. Survey data were collected in June 2023. All study procedures were approved by the Regional Research Ethics Committee of Qassim Province and the Institutional Review Board of the Johns Hopkins Bloomberg School of Public Health.

### Participants

Our target sample was individuals who read/speak Arabic and reside in Saudi Arabia, including potential stakeholders such as policymakers, information technology industry leaders, clinicians, academic researchers, educators, regular and non-regular digital media users, parents, adolescents, and young adults (18 years to 24 years).

### Measures

The questionnaire was delivered using SurveyMonkey and included questions derived from the investigators’ prior work [5] and the Saudi Health Interview Survey [21]. Questions were translated from English to Arabic and then back-translated by two different bilingual researchers. The online questionnaire was pretested by six bilingual university students who provided feedback on question clarity and response options. After pretesting, we added definitions for unfamiliar terms such as “digital wellbeing”, shortened questions to focus on the primary device/activity, eliminated redundancies by deleting repetitive questions across sections, and adjusted Arabic wording for improved understanding and readability [22].

The questionnaire assessed stakeholder demographic characteristics, such as age, gender, work status, province, and marital status. Additionally, participants chose a primary identity for the purposes of representation and coverage (Table 1), for example: regular digital media users, individuals with problematic internet use, professional belonging to different groups, policymakers, gamers, and others. The questionnaire also asked about digital media use and experiences, such as relative importance of digital media activities (e.g., social networking websites; apps for chatting, messaging, or video chatting; email; games; etc.), reasons for use (e.g., to relax; to achieve or learn something; to forget about unpleasant things; etc.), and effects of use (mainly positive, mainly negative, no influence) on various domains (e.g., concentration; school, study, or work; contact with family and friends; sleep; etc.). Moreover, the questionnaire included questions about the desire to regulate their own digital media use (yes/no) and types of actions taken to regular use (e.g., “digital detox” or break; set clear goals; deleted apps; blocked websites; etc.). Finally, the questionnaire explored perspectives on the most important target population and the potential success of specific types of interventions to improve digital wellbeing in Saudi Arabia. Several of the questions included an “other” response option and allowed participants to enter a free text response. A copy of the full questionnaire in English translation can be found in the supplementary material.

### Data analysis

We examined distributions of categorical variables and collapsed categories for questions related to participant income, media use reasons, media use time, and perceived likelihood of success of digital wellbeing interventions. For example, response options for questions about digital media use reasons indicating ‘never’ or ‘almost never’ were recoded into one category (i.e., ‘never/almost never’), and responses of ‘usually’ or ‘often’ were recoded as well (i.e., ‘usually/often’). Media use time responses were also combined into broader hourly categories (i.e., less than 2 h; 2 to less than 4 h; 4 to less than 6 h; 6 h or more) to facilitate clearer and more streamlined reporting. Additionally, participant perspectives on perceived likelihood of success of digital wellbeing interventions were categorized from 5 response options (extremely unlikely; unlikely; neutral; likely; extremely likely) into 3 likelihood groups: ‘unlikely’, ‘neutral’, and ‘likely’. We also created a stakeholder group variable to condense stakeholders’ primary group identification (Table 1). One participant missed group identification and was not included in these groupings. All analyses were conducted using R. Mean rank, standard deviation, and percentage were calculated for the choices of each ranking question.

## Results

### Sample description

Ninety-two people comprised of 13 stakeholder groups completed the survey (Table 1). Most were male (63.0%) and under 25 years old (46.7%). For the primary stakeholder identity question (Table 1), participants identified primarily as regular digital media users (26.1%), persons with problematic Internet use (18.5%), or clinicians/healthcare professionals (18.5%). The majority of participants were from Qassim (45.7%), Riyadh (25.0%), and the Eastern Province (9.8%).

#### Media use experiences and reasons for use

Generally, participants reported digital media use between 6 and 10 h a day. Most applicants ranked social networking and communication apps as their most frequently used (Table 2), whereas content creation and “other” were ranked as the least used.

**Table 2:** Media activity rankings

Media Activity	Percent Rank #1	Percent Rank #2	Percent Rank #3	Mean Ranking	SD
Social networking	36.0	32.0	25.0	2.1	1.6
Apps for chatting	40.0	27.0	14.0	2.4	2.0
Streaming/watching videos	8.0	10.0	18.0	4.6	2.3
Email	2.0	11.0	13.0	5.3	2.5
Playing games on phone	2.0	3.0	11.0	5.7	2.4
Browsing internet	4.0	7.0	8.0	5.8	2.5
Playing games on console/laptop	5.0	8.0	4.0	6.3	3.0
Listening to music/podcasts/other media	1.0	1.0	4.0	8.5	3.1
Online shopping	2.0	1.0	1.0	8.8	3.0
Programming	0.0	1.0	0.0	9.2	2.1
Creating vlogs/videos	0.0	0.0	1.0	9.6	1.9
Creating music/blogs/other	0.0	0.0	0.0	10.4	1.5
Other	0.0	0.0	0.0	12.3	1.4

Mean Ranking: the perceived importance of each activity, with lower values indicating higher perceived importance

Percent Rank #: refers to the ranking order assigned to each media activity based on the percentage of respondents who ranked them within their top three preferences

*SD* Standard Deviation

**Fig. 1:**
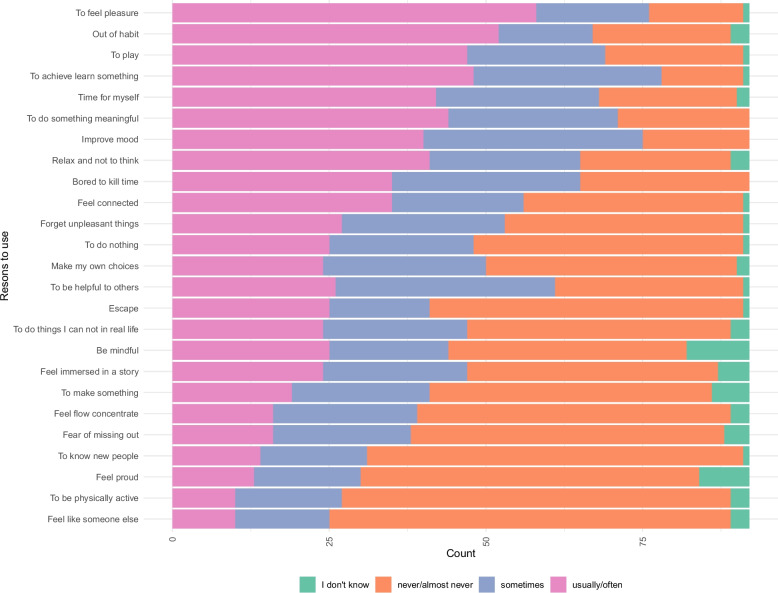
Distribution of the reasons for individuals’ preferences for digital media use

Participants reported various reasons for using their top media choice (Fig. 1).

Most participants chose pleasure, habit, playfulness, or learning/achieving something as reasons they usually or often chose to use their top choice of digital media. Moreover, participants rarely endorsed feeling like someone else, being active, or feeling proud as reasons for use. The time spent on digital media differed slightly between work or school and recreational use (Table 3).

**Table 3:** Amounts of digital media/screen use

Use category	Amount of use				
	less than 2 h	2 to less than 4 h	4 to less than 6 h	6 h or more	Don’t know/can’t say
Use for work or school: weekday	38.0%	25.0%	19.6%	14.1%	3.3%
Use for work or school: weekend	33.7%	27.2%	16.3%	19.6%	3.2%
Recreational use: weekday	38.1%	13.0%	27.2%	20.6%	1.1%
Recreational use: weekend	22.8%	18.5%	20.7%	35.9%	2.1%
TV watching	79.4%	8.7%	7.6%	3.3%	1.0%

For work or school digital media use, about a third of participants reported 4 or more hours a day of daily use (33.7% on weekdays; 35.9% on weekends). For recreational use, about half of the participants reported 4 + hours of daily use (47.0% on weekdays; 56.6% on weekends). Of note, 36.9% of participants reported 6 h or more of digital media use for recreation on weekends.

#### Perceived effects of digital media use

**Fig. 2:**
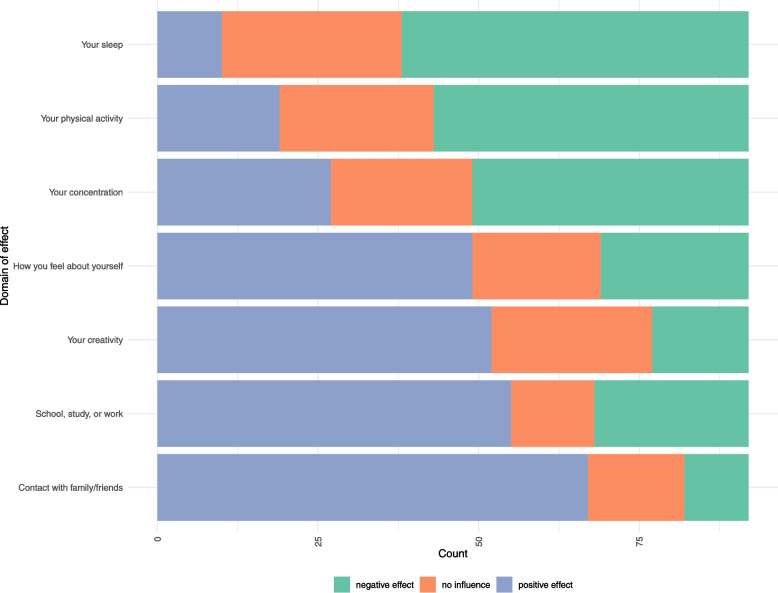
Distribution of the perceived effects of digital media use on different domains of life

Participants generally felt that using their top media choice had a positive effect on their ability to stay in touch with family and friends, performance in school or at work, and creativity (Fig. 2).

More than 50.0% reported that this had mainly a positive effect on how they felt about themselves. However, over half of the participants reported that media use negatively affected sleep and physical activity.

#### Self-regulation of digital media use

Most of the participants (72.8%) reported wanting to regulate their digital media use. Of these participants (Table 4), most chose to abstain by either deleting an app from their phone (45.0%) or taking a “digital detox” or break period (37.0%).

**Table 4:** Approaches to self-regulation of use (*n* = 70, choose all that apply)

Method	Count	%^a^
I’ve deleted an app from my phone	45	64.3
I’ve taken a “digital detox” or break period	37	52.9
I’ve set a fixed bedtime or other time of day where I stop	31	44.3
I’ve planned the time of day I use digital media–I’ve worked it into my schedule	30	42.9
I’ve set clear goals	29	41.4
I’ve put informal limits on the amount of time I use digital media	26	37.1
I’ve set an alarm	22	31.4
I’ve set notifications in an app, on the computer or on a game	19	27.1
I’ve chosen only social types of media that require others (e.g., partners, team members) to be online at the same time	19	27.1
I’ve asked someone to help me limit my use	10	14.3
I’ve used an app on my smartphone	11	15.7
I’ve used parental control features or another feature that limits my time online or on a specific app or game	10	14.3
I’ve sold or gotten rid of a videogame	10	14.3
I’ve set limits for money spent on digital media	6	8.6
I’ve used an app or website on my computer	7	10.0
I haven’t taken any formal actions	6	8.6
I’ve blocked a website	7	10.0
I haven’t taken any formal actions, but have thought about it	4	5.7

^a^This question allowed respondents to choose more than one answer and was calculated out of those who answered that they had ever tried to regulate their own use. (*n* = 70)

Focusing on time management for self-regulation, such as creating schedules or setting time limits, was also popular. Fewer participants reported using apps to control their use or blocking websites. In the “other” text response, some participants recommended other strategies (e.g., “I watched tips on YouTube”), while others relied on their own self-control abilities or faith (e.g., “I committed myself ‘by swearing in God’s name’ not to open a certain app for a week or a month.”).

#### Perspectives on interventions to improve digital wellbeing in Saudi Arabia

**Fig. 3:**
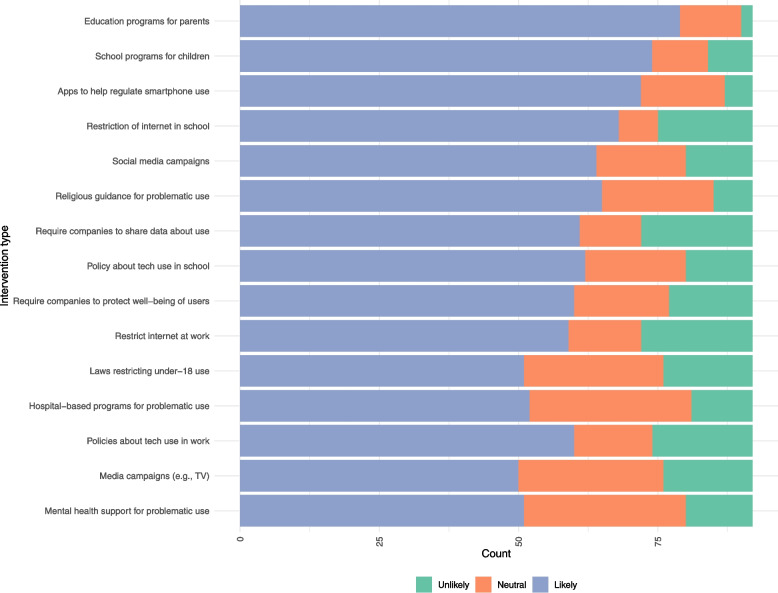
Distribution of perceived effectiveness of various interventions for improving digital wellbeing in Saudi Arabia

Stakeholders indicated their agreement for the potential success of 15 types of interventions to improve digital wellbeing in Saudi Arabia (Fig. 3).

Participants generally felt all interventions were likely to work (i.e., “likely” was endorsed by 50.0% of participants or more), with education-based interventions (*programs for parents* and *school programs for children*) and *apps to help regulate smartphone use* being endorsed by at least 75.0% of participants. Participants rarely thought interventions were unlikely to work; only two interventions (*require companies to share data about use* [21.7%] and *restricting internet at work* [21.7%]) were endorsed as unlikely by more than 20.0% of the participants. Stakeholders also ranked the potential targets of interventions: adolescents (mean rank 2.2), school children (mean rank 2.6), and young adults (mean rank 2.8) were ranked as the ideal population targets (Table 5).

**Table 5:** Suggested population targets for intervention

Group	Percent Rank #1	Percent Rank #2	Percent Rank #3	Mean Ranking	SD
Adolescents (13 to 17)	33.7	25.0	29.3	2.2	1.2
School children (6 to 12)	18.5	33.7	25.0	2.6	1.2
Young adult (18 to 25)	22.8	21.7	19.6	2.8	1.3
Young children (age < 6)	21.7	9.8	13.0	3.5	1.8
Adults (26 to 55)	3.3	8.7	8.7	4.3	1.2
Older adults (age > 56)	0.0	1.0	4.3	5.6	0.85

Mean Ranking: the perceived importance population to target, with lower values indicating higher perceived importance

Percent Rank #: refers to the ranking order assigned to each population group based on the percentage of respondents who ranked them within their top three preferences

*SD* Standard Deviation

## Discussion

We conducted a survey of 92 stakeholders to inform the development of culturally tailored programs to improve digital wellbeing in Saudi Arabia. Our participants generally reported high digital media use (between 6 and 10 h a day), with 18.5% identifying primarily as individuals with problematic use. Participants reported differential impacts of their digital media/technology use ranging from positive (e.g., contact with family and friends, performance at school/work) to negative (e.g., disrupted sleep, reduced physical activity). Participants generally felt that many intervention strategies could be successful in improving digital wellbeing in Saudi Arabia, the most highly rated among them being education programs for parents and school programs for children. Previous studies have demonstrated that interventions to reduce screen time delivered to parents may have greater effects than those given to children and parents or children alone [23]. Given the strong collectivist culture and family values in Saudi Arabia, interventions focusing on students and parents would likely need to consider these values [24, 25].

Participants also highly endorsed social media campaigns to educate and raise awareness about digital technology use and digital wellbeing, while campaigns using traditional media (e.g., TV) were ranked less highly. Although media campaigns to improve digital wellbeing have been implemented in national settings (e.g., Ithra Sync [26], Digital Balance by Trimbos & Netwerk Mediawijsheid in The Netherlands [27]), evaluations of these campaigns have not been conducted. One published study of a mail-based campaign -a pamphlet providing parenting strategies to prevent Internet gaming disorder (intervention) mailed to a representative sample of parents in Norway -showed no differences in children’s Internet gaming disorder symptoms or video gaming use, compared to a control group (no intervention) [28]. Formal testing of various types of health promotion campaigns to promote digital wellbeing is required to improve their evidence base.

There are some overall tensions between education programs that focus on individual users compared to policy regulations that impact populations at large, and these tensions are visible in the findings of the current study. On the one hand, participants’ high endorsement of approaches that raise awareness and educate users underscore the importance of positive messaging from a media literacy perspective. Mass media messaging, as a health behavior change approach, has mixed effects for various types of interventions, with actual behavior change outcomes being dependent on several factors [29]. Approaches that empower individuals to make informed decisions and exercise self-regulation (i.e., interventions containing goals, feedback, and planning) have been shown to be more effective for screen time reduction than interventions without these components [23].

On the other hand, our study found mixed results for participant beliefs in the utility of different policies and restrictions to improve digital wellbeing. For example, restrictions on Internet use in schools were rated relatively highly (73.9%), while laws restricting digital media use of individuals under 18 years old were ranked relatively low (55.4%). Perspectives on the role of the tech industry in shaping the population-level use of digital technology have affected the discourse on the importance of user self-regulation vs. policy. For example, the industry has long employed narratives of end user responsibility to avoid regulation [4], which may be reflected in participant responses of potential ineffectiveness of policies. Elucidation of dark patterns in commercial software platforms has led to a better understanding of the ways in which software design reduces user autonomy and promotes over-engagement [30]. Existing research shows some indications of the mixed success of policy restrictions. While we know that shutdown laws may not be very effective in reducing adolescents’ internet use [31], newer rules (e.g., Consumer Protection in Online Video Games: A European Single Market Approach, 2023 [32]), highlight the necessity for the industry take actions to protect consumers. This suggests that putting the onus on industry to protect individuals might work better than just restricting individual use. Experience from tobacco control and regulation of alcohol globally has demonstrated that population-level impact to protect public health can be expected from strategies that restrict the affordability, availability, and marketing of these products [33, 34]. In Saudi Arabia, the taxation of sugar sweetened beverages [35], a ban on artificial trans-fats [36] and the implementation of taxation and plain packaging on tobacco products [37, 38] demonstrate the potential of successful regulatory strategies. However, given the role of digital media in everyday life, outright bans seem unrealistic. Therefore, a multi-mode approach that uses education and limitations targeting the most problematic digital media use might be a strategy for future consideration.

Our study found that religious guidance for individuals who need help in controlling their digital media use was also supported as a strategy to improve digital wellbeing. Incorporating aspects of digital wellbeing into religious education and communication may help prepare Saudi users to resist challenges brought on by technology designed to encourage excessive use. Promotion of moderation is a cultural value that could be leveraged in digital wellbeing interventions in this setting. Formative research in the US Muslim community suggests that religiously-tailored messages may be useful in overcoming barriers to health behavior change to promote screening mammograms [39]. Continuing stakeholder engagement could be useful to refine the framing and content of health behavior change messaging in future interventions and address potential ethical challenges.

Participants in the current study reported they felt a positive impact of digital media use in several domains, including contact with friends and family, use of technology for school, study or work, creativity, and how they feel about themselves. On the other hand, participants reported that digital media negatively affected their sleep, physical activity, and concentration. Similar to our findings, several studies highlight the detrimental impacts of prolonged screen time on the quality and duration of sleep and increased sedentary time, particularly among children and adolescents [40–42]. This underscores the importance of encouraging behavior that enhances digital well-being. Based on our findings, interventions that help individuals increase physical activity, enhance sleep hygiene, and buffer the negative impacts of technology on concentration may be most successful.

Stakeholders in the current study suggested adolescents, school children, and young adults as priority populations for digital wellbeing interventions in Saudi Arabia. Moreover, a substantial proportion of stakeholders reported their own experience of problematic internet use as their primary stakeholder group identity (18.5%). These findings are aligned with recent concerns about problematic internet use, internet addiction, and negative impacts on mental health and wellbeing, especially among young people in Saudi Arabia [2]. Participants in our study rated hospital-based programs and mental health support for problematic use relatively low among other potential interventions to improve digital wellbeing. This low endorsement of hospital and mental health settings as effective supports could reflect little confidence in these types of interventions. Alternatively, cultural attitudes that reflect persistent mental health stigma and a preference to handle problems individually may also play a role [43–45]. Thus, it will be vital to address clinically-relevant problematic technology use in future research and ensure that digital media use problems and negative consequences are destigmatized, and access to treatment is provided.

### Strengths and limitations

The findings of the current study should be interpreted with several limitations in mind. First, fewer than 100 stakeholders were recruited using a targeted sampling strategy through known networks of investigators and collaborators. Therefore, some stakeholder groups were represented in low numbers (e.g., industry members, legal professionals, religious leaders). This may have resulted in limitations to the generalizability of the sample and study findings. We aimed for geographic diversity, but due to logistical constraints, national representation was not fully achieved. Most participants were from Qassim, Riyadh, and the Eastern Province, with limited representation from the northern and southern regions. However, the inclusion of stakeholders from key central provinces offers useful preliminary insights. Future studies with larger and representative samples are warranted that could use more robust inferential statistics and conduct subgroup analyses for key stakeholder groups. Moreover, follow up studies utilizing interviews and focus groups with stakeholders are needed to provide in-depth data to inform the development of interventions to improve digital wellbeing in Saudi Arabia. The current study used self-reported measures that may be impacted by social desirability bias, and we did not include objective measures of digital media use. Finally, the limited sample size, the potential overrepresentation of certain stakeholder groups such as educators and clinicians, and the instruments used may have influenced the conclusions drawn; thus, caution is warranted when generalizing these findings. Strengths of our study included that we purposefully recruited stakeholders from various groups in society that presented a range of different perspectives on digital technology use. Moreover, we included questions to assess both benefits and drawbacks of technology use, to provide a comprehensive perspective on the role of technology in Saudi Arabia.

## Conclusions

Findings collected from diverse stakeholders suggest that interventions to improve digital wellbeing for the Saudi Arabian context should focus on young people and potentially combine approaches focusing on education and promotion of self-regulation of digital media use (e.g., school-based programs for children/adolescents and parents, apps to help people self-regulate use). While not as highly rated by stakeholders, policies restricting specific aspects of technology use and overuse and requiring companies to protect users’ wellbeing could also be considered for population-level impact. As Saudi Arabia aims to become a leader in the global knowledge economy, digitalization, and entertainment sectors, it is crucial to involve relevant parties in creating strategies that are both culturally appropriate and practical, which will help maintain a healthy digital lifestyle for its society. Future studies could build on the findings reported in the current study, expand sampling of stakeholder groups and thus improve generalizability of findings, include objective measures of digital media use, and conduct qualitative work with key stakeholders to give findings greater context and depth. Moreover, future work is needed to develop and test interventions for improving digital wellbeing in Saudi Arabia, and the current study can be an important step in that direction.

Future research should aim to include a more balanced and representative sample to enhance the validity and applicability of the results. As a next step, we are developing intervention components with stakeholder input through workshops, followed by pilot testing in educational and family settings. This process includes iterative feedback from youth, educators, and policymakers to ensure the intervention is culturally appropriate, acceptable, and feasible. Insights from these stages will inform a school-based randomized controlled trial in Saudi Arabia.

## Supplementary Information

Supplementary Material 1.

## Data Availability

The dataset used during the current study isavailable from the corresponding author upon reasonable request.
