# Supplementary material for: Stakeholder perspectives on digital wellbeing in Saudi Arabia: a cross-sectional survey
Source: BMC Public Health. 2025 Jul 14;25:2451. doi: 10.1186/s12889-025-23674-4 (PMC12257859; doi:10.1186/s12889-025-23674-4)
Supplement: Supplementary file 1 — Supplementary Material 1. [file 12889_2025_23674_MOESM1_ESM.docx]

# **Stakeholder survey**

Welcome back to the Digital Wellbeing in Saudi Arabia study, conducted by Johns Hopkins Bloomberg School of Public Health in collaboration with Sulaiman Al Rajhi University.

Thank you for completing phase 1.

In phase 2, we hope to engage you as our stakeholder and we request you to answer the following questions to the best of your ability. We appreciate your input and opinions. This survey should take about 15 minutes and we will send you a gift coupon for 100 Riyals via Whatsapp after completing the survey.

Please click here for your agreement and to begin the survey.

## Block 1: Definitions

**What do you think of when you think of digital media use or screen time? What types of activities do you see as being a part of this term? [paragraph text entry]**

[Show definition] For this survey, we will define digital media use/screen time as including social media (e.g. Twitter, Facebook, TikTok, Instagram), smartphones (e.g., iPhone or Android), tablets (e.g., iPad), the Internet (going online on a computer or a device), communication apps (e.g., WhatsApp), streaming entertainment and videos (e.g., YouTube, Netflix) and videogames played on a computer, console, or mobile phone.

## Block 2: Digital media use habits

**On which type of digital media do you spend the most time in your free time? This refers to using digital media for recreation rather than for work, school, or running a business or household.**

**Please order the following media activities by the amount of time you spend on them, so that the one you spend the most time on is ranked first. For example, if you spent most of your time on digital media watching TV, you would [move that to the top or whatever the survey platform’s mechanism is].**

**[For interviews only]:If you’d like, you can check your phone to see if there’s a feature that helps track the amount of time you spend on different apps (e.g., Apple/iOS/Android Screen Time app).**

Apps for chatting, messaging or video chatting like WhatsApp, Snapchat or Facebook Messenger

Social networking websites like Facebook, Twitter, Instagram, TikTok or Pinterest

Email (on a computer, laptop, mobile phone or tablet)

Games on a mobile phone or tablet

Games on a computer, laptop or console

Watching: Series, movies or videos (for example, on TV, in the movie theater, on Netflix or Youtube)

Browsing, looking for information and reading websites on digital media (for example, online newspapers or magazines)

Programming

Creating videos or vlogs

Creating something else using a computer, for example music or blogs

Listening to music, podcasts, audiobooks, or some other type of audio media

Shopping online

Something else, specifically: _____

**We’d like to understand why people spend time on their preferred tech choices.**

**Please answer the following questions on why you spend time on your primary digital media activity.**

|  | Not at all | Almost never | Sometimes | Usually | Often | I don’t know |
| --- | --- | --- | --- | --- | --- | --- |
| To relax and not have to think about things for a moment |  |  |  |  |  |  |
| To concentrate or get into “the flow” |  |  |  |  |  |  |
| To achieve or learn something |  |  |  |  |  |  |
| To do something meaningful |  |  |  |  |  |  |
| To do something for someone else |  |  |  |  |  |  |
| For pleasure |  |  |  |  |  |  |
| To make my own choices |  |  |  |  |  |  |
| To be proud and satisfied |  |  |  |  |  |  |
| To have time for myself |  |  |  |  |  |  |
| To be immersed in a story |  |  |  |  |  |  |
| To do nothing at all |  |  |  |  |  |  |
| To feel connected to others |  |  |  |  |  |  |
| To make something (digital or physical) |  |  |  |  |  |  |
| To be mindful/be aware of my thoughts and feelings |  |  |  |  |  |  |
| To be playful and play |  |  |  |  |  |  |
| To be physically active |  |  |  |  |  |  |
| To get to know new people |  |  |  |  |  |  |
| To forget about unpleasant things |  |  |  |  |  |  |
| To get into a better mood |  |  |  |  |  |  |
| To do things I can’t do in offline life (ie, IRL) |  |  |  |  |  |  |
| To escape from real life |  |  |  |  |  |  |
| To feel as if I were somebody else for a while |  |  |  |  |  |  |
| It’s just a habit-I start doing it without thinking |  |  |  |  |  |  |
| Because I’m bored and just want to kill time |  |  |  |  |  |  |
| Because I’m worried I’ll miss out on what’s happening |  |  |  |  |  |  |

## Block 3: Amount of digital media use

**Now let’s think about using digital media/screens. This includes social media (e.g., Twitter, Facebook, TikTok, Instagram), smartphones (e.g., iPhone or Android), tablets (e.g., iPad), the Internet (going online on a computer or a device), communication apps (e.g., WhatsApp), streaming entertainment and videos (e.g., YouTube, Netflix) and videogames played on a computer, console, or mobile phone.**

**Think about a typical weekday, that is, a day when you are working, going to school, or taking care of the household. This is a day that you’re busy—not a day to relax. What would you say is your total amount of screen time digital media use that is just for work, school, or taking care of the household?**

o None

o Less than ½ hour

o ½ hour to less than 2 hours

o 2 hours to less than 4 hours

o 4 hours to less than 6 hours

o 6 hours to less than 8 hours

o 8 hours to less than 10 hours

o 10 hours to less than 12 hours

o 12 hours or more

o Don’t know

o I cannot say

**Now think about a typical weekend day, or another day when you don’t have work or school (or a day when you have few household responsibilities if you’re a homemaker)—a free day. Do you use screens for work, school, or household responsibilities on the weekend? If so, what would you say is your total amount of screen time or digital media use that is just for work, school, or household management?**

o None

Less than ½ hour

o ½ hour to less than 2 hours

o 2 hours to less than 4 hours

o 4 hours to less than 6 hours

o 6 hours to less than 8 hours

o 8 hours to less than 10 hours

o 10 hours to less than 12 hours

o 12 hours or more

o Don’t know

o I cannot say

**Now let’s think about using digital media/screens for your own purposes—for recreation or entertainment--outside of work or school. On a typical weekday, how much time do you usually spend in front of the television, videogames or on the computer or in front of a digital device such as a smartphone or tablet that is not work, school or household-management related?**

o None

Less than ½ hour

o ½ hour to less than 2 hours

o 2 hours to less than 4 hours

o 4 hours to less than 6 hours

o 6 hours to less than 8 hours

o 8 hours to less than 10 hours

o 10 hours to less than 12 hours

o 12 hours or more

o Don’t know

o I cannot say

**On a typical weekend day, how much time do you usually spend in front of the television, videogames or on the computer or in front of a digital device such as a smartphone or tablet that is not work, school, or household-management related?**

o None

Less than ½ hour

o ½ hour to less than 2 hours

o 2 hours to less than 4 hours

o 4 hours to less than 6 hours

o 6 hours to less than 8 hours

o 8 hours to less than 10 hours

o 10 hours to less than 12 hours

o 12 hours or more

o Don’t know

o I cannot say

**Specifically, about how much time do you spend watching TV each day?**

o None

Less than ½ hour

o ½ hour to less than 2 hours

o 2 hours to less than 4 hours

o 4 hours to less than 6 hours

o 6 hours to less than 8 hours

o 8 hours to less than 10 hours

o 10 hours to less than 12 hours

o 12 hours or more

o Don’t know

o I cannot say

**Does the use of a digital media mainly have a positive or mainly a negative effect on:**

[Answer options: Mainly a positive effect, Mainly a negative effect, No influence.]

|  | Mainly a positive effect | Mainly a negative effect | No influence |
| --- | --- | --- | --- |
| Your concentration |  |  |  |
| School, study or work |  |  |  |
| How do you feel about yourself |  |  |  |
| Contact with family and friends |  |  |  |
| Your sleep |  |  |  |
| The extent to which you exercise or are physically active |  |  |  |
| Your creativity |  |  |  |

## Block 4: Self-regulation of digital media use

Have you ever wanted to regulate your digital media use?

Yes

No

Pipe to (if yes)

You answered that you’ve wanted to regulate your media use. What types of actions have you taken to regulate your use? Check all that apply.

1. I’ve taken a “digital detox” or break period
2. I’ve asked someone to help me limit my use
3. I’ve chosen only social types of media that require others (e.g., partners, team members) to be online at the same time
4. I’ve planned the time of day I use digital media--I’ve worked it into my schedule
5. I’ve set clear goals
6. I’ve put informal limits on the amount of time I use digital media
7. I’ve used parental control features or another feature that limits my time online or on a specific app or game
8. I’ve set limits for money spent on digital media
9. I’ve deleted an app from my phone
10. I’ve sold or gotten rid of a videogame
11. I’ve blocked a website
12. I’ve set notifications in an app, on the computer or on a game
13. I’ve set an alarm
14. I’ve set a fixed bedtime or other time of day where I stop
15. I’ve used an app on my smartphone
16. I’ve used an app or website on my computer
17. I’ve done something else [write it]
18. I haven’t taken any formal actions, but have thought about it
19. I haven’t taken any formal actions

How has your experience been with your self-regulation strategies?

[write in]

## Block 5: Physical activity

**Now we are going to ask you about the time you spend doing different types of physical activity in a typical week, whether for work, travel, or leisure/recreation. Please answer these questions even if you do not consider yourself to be a physically active person.**

In answering the following questions, ‘vigorous intensity activities' are activities that require hard physical effort and cause large increases in breathing or heart rate; 'moderate-intensity activities' are activities that require moderate physical effort and cause small increases in breathing or heart rate. These can be exercise or sports, but can also just be time spent with friends playing active games, getting to and from work by walking or cycling, or other things you can think of.

**A low level of physical activity is considered as a total of less than 150 minutes of moderate to vigorous activity per week. A moderate level of physical activity is considered as a total of 150–300 minutes of moderate to vigorous activity per week. A high level of physical activity is considered as a total of more than 300 minutes of moderate to vigorous activity per week.**

**Please rate your level of physical activity in a typical week.**

o Not active

o Low activity (less than 150 minutes/week)

o Moderately active (150–300 minutes/week)

o Highly active level (more than 300 minutes/week)

o Don’t know

o I cannot say

**The following question is about sitting or reclining, whether you’re at work, at home, getting to and from places, alone or with someone. This includes time spent sitting at a desk, sitting with friends, traveling in car, bus, train, reading, playing cards, or using digital media but do not include time spent sleeping.**

About how many hours do you spend sitting or reclining per day?

o None

Less than ½ hour

o ½ hour to less than 2 hours

o 2 hours to less than 4 hours

o 4 hours to less than 6 hours

o 6 hours to less than 8 hours

o 8 hours to less than 10 hours

o 10 hours to less than 12 hours

o 12 hours or more

o Don’t know

o I cannot say

## Block 6: Perspectives on research and intervention on digital wellbeing

**Now we’re going to discuss digital wellbeing, which we define as living a healthy life in a digital world, using digital media and technologies to enhance and improve our lives.**

If the overall goal is to improve digital wellbeing and help people get the best use of digital media and technology, what do you think should be done? That is, what kind of program or change would make a difference in Saudi society? **[write in]**

**Who do you think would benefit the most from strategies to improve digital wellbeing in Saudi society?**

Young children (age < 6)

School children (6 to 12)

Adolescents (13 to 17)

Young adults (18 to 25)

Adults (26 to 55)

Older adults (age >56)

**Please add any other specific groups that you think might benefit from strategies to improve digital wellbeing in Saudi society? (Examples: married people, healthcare professional, or students, etc) [write in]**

**How likely do you think the following types of interventions would be to have a positive impact on digital wellbeing in Saudi society?**

**Response scale
Extremely Unlikely – Unlikely – Neutral – Likely – Extremely Likely**

1. Laws restricting internet use by people under 18
2. Policies in schools about use of technology by students
3. Policies in workplaces about use of technology by employees
4. Restriction of specific internet sites in schools
5. Restriction of specific internet sites in workplaces
6. Requirements that software companies and platforms take actions to protect their users’ digital wellbeing (e.g., by reducing things that make their products “addictive”)
7. Requirements that software companies share data about how much time people spend on their products
8. Hospital-based programs for people who need help controlling their digital media use
9. Mental health support (e.g., hotline) for people who need help controlling their digital media use
10. Religious guidance for people who need help controlling their digital media use
11. Media campaigns (e.g., on TV) about digital wellbeing
12. Social media campaigns (e.g., Twitter) about digital wellbeing
13. School programs for children to teach digital wellbeing
14. An app to help people regulate their smartphone use
15. Educational programs to help parents learn how to parent in a digital age

**Thinking about different ways we can change people's technology and use behavior to improve digital wellbeing, are there any options that will NOT work in Saudi society?**

[Write in]
